# Shared Risk Factors and Molecular Mechanisms Between Aortic Stenosis and Atherosclerosis: A Rationale for Therapeutic Repositioning

**DOI:** 10.3390/ijms26178163

**Published:** 2025-08-22

**Authors:** Corina Cinezan, Dan Claudiu Magureanu, Maria Luiza Hiceag, Camelia Bianca Rus, Ioana Tiberia Ilias, Iulia Denisa Bogdan, Alexandra Manuela Buzle, Angela Cozma

**Affiliations:** 1 Department of Medical Disciplines, Faculty of Medicine and Pharmacy, University of Oradea, 410073 Oradea, Romania; rus.cameliabianca@student.uoradea.ro (C.B.R.); ioana.ilias@didactic.uoradea.ro (I.T.I.); bogdan.iuliadenisa@student.uoradea.ro (I.D.B.); buzle.alexandramanuela@student.uoradea.ro (A.M.B.); 2 Clinical County Emergency Hospital Bihor, 410169 Oradea, Romania; 3 Department of Pharmacology, Toxicology and Clinical Pharmacology, Iuliu Hatieganu University of Medicine and Pharmacy, 40337 Cluj-Napoca, Romania; 4 Cardiology Department, Niculae Stancioiu Heart Institute, 400001 Cluj-Napoca, Romania; 5 Cardiology Department, Rehabilitation Hospital, 400347 Cluj-Napoca, Romania; maria.luiz.hiceag@elearn.umfcluj.ro; 6 Cardiology Department, Municipal Hospital Aiud, 515200 Aiud, Romania; 7 Doctoral School of Biological and Biomedical Sciences, University of Oradea, 410087 Oradea, Romania; 8 4th Department of Internal Medicine, Faculty of Medicine, Iuliu Hatieganu University of Medicine and Pharmacy, 400012 Cluj-Napoca, Romania; angela.cozma@umfcluj.ro

**Keywords:** aortic stenosis, atherosclerosis, risk factors, mechanism, PCSK 9 inhibitors, lipoprotein(a), DPP-4 inhibitors, denosumab, therapeutic repositioning

## Abstract

Aortic stenosis (AS) and atherosclerosis are progressive cardiovascular conditions that frequently coexist and share multiple clinical and molecular features. Medical therapies have shown effectiveness in preventing and treating atherosclerosis and its consequences. For AS, effective pharmacological therapies remain limited. Understanding the shared risk factors and mechanisms between the two conditions may provide opportunities for therapeutic repositioning in AS. We performed a narrative review focusing on studies published from 2005 to 2025. Inclusion criteria encompassed clinical trials, experimental models, and molecular studies addressing overlapping risk factors, pathological pathways, and treatment approaches for AS and atherosclerosis. AS and atherosclerosis share key risk factors, including age, hypertension, hyperlipidemia, and diabetes. Molecular mechanisms, such as chronic inflammation, endothelial dysfunction, oxidative stress, lipid accumulation, and calcific remodeling, are common to both. Pathways involving the renin-angiotensin system, Notch signaling, and osteogenic mediators contribute to disease progression. Several drug classes, notably proprotein convertase subtilisin/kexin type 9 (PCSK9) inhibitors, lipoprotein(a) (Lp(a)) lowering therapies, anti-inflammatory agents, and immunomodulators, show potential for repositioning in AS management. The substantial overlap in risk factors and molecular mechanisms between AS and atherosclerosis supports a rationale for therapeutic repositioning. Targeting shared pathways could lead to innovative strategies for slowing AS progression and improving patient outcomes.

## 1. Introduction

Valvular heart disease affects millions of individuals worldwide and contributes substantially to cardiovascular morbidity and mortality. Among valvular pathologies, aortic stenosis is the most prevalent form of valvular heart disease in developed countries, affecting approximately 2–3% of adults over 65 years of age and increasing to nearly 10% in those over 80 years [1]. Aortic stenosis (AS) is a progressive disease, leading to valvular calcification with impaired leaflet mobility and ultimately to heart failure if left untreated. Traditionally considered a passive degenerative process resulting from aging and mechanical stress, contemporary research has redefined AS as an active disease [2].

Aortic stenosis and atherosclerosis, particularly coronary artery disease, frequently coexist in elderly individuals, largely due to their shared risk factors. Over the years, it has been emphasized that these risk factors are present in patients with calcific aortic stenosis, even in the absence of coronary artery disease. Thus, the idea of a common molecular mechanism of the two disorders emerged, aortic stenosis being a cell-mediated disease sharing numerous pathological features with atherosclerosis [3,4].

AS and atherosclerosis are both characterized by endothelial dysfunction, lipid accumulation, chronic inflammation, and progressive calcification. In AS, mechanical stress and endothelial damage lead to the infiltration of lipids and inflammatory cells into the aortic valve, initiating a cascade of events that promote calcification [5]. Similarly, atherosclerosis involves the accumulation of lipids within the arterial wall, triggering inflammatory responses that contribute to plaque formation and vascular calcification [6].

Keeping in mind that, like atherosclerosis, aortic stenosis is a progressive disease and can be a devastating one, leading to heart failure and death, researchers tried to find therapeutic approaches to slow its progression. Despite the shared mechanisms of the two diseases, pharmacological interventions effective in atherosclerosis, such as statins and some anti-inflammatory agents, have not demonstrated significant efficacy in halting the progression of AS in clinical trials [7]. This discrepancy underscores the need for a deeper understanding of the molecular pathways common to both diseases to identify potential therapeutic targets.

This narrative review is focused on elucidating the shared molecular and cellular mechanisms underlying AS and atherosclerosis, drawing attention to the common risk factors. It also critically assesses the current evidence on the efficacy of anti-atherosclerotic therapies in AS and explores the potential for therapeutic repositioning based on these common pathways.

## 2. Materials and Methods

We conducted a comprehensive literature search using PubMed, Scopus, and Web of Science to identify relevant studies published between 2005 and 2025. Keywords included “aortic stenosis,” “atherosclerosis,” “shared risk factors,” “valvular calcification,” “inflammation,” “lipoprotein(a),” “PCSK9,” “interleukin-6,” and “molecular mechanisms.” Boolean operators (AND, OR) were used to combine search terms. We included original research articles, reviews, and clinical trial data published in English that addressed epidemiological, molecular, and therapeutic aspects common to both diseases. References from key articles were manually screened to identify additional relevant sources. Priority was given to studies with mechanistic insights, large cohort data, and evidence from randomized controlled trials. Articles focusing solely on congenital valve disease or infectious etiologies were excluded.

## 3. Risk Factors

There is a wide variety of risk factors that contribute to aortic valve calcification, many of these factors being associated with atherosclerosis. Since aortic valve calcification is the most common acquired valvular disease, and atherosclerosis remains the leading cause of death in developed countries, there is considerable interest in understanding the risk factors contributing to this condition and its similarities to atherosclerosis. Risk factors can be classified into non-modifiable and modifiable [1,8].

The non-modifiable risk factors include age, gender, and family history. Age is one of the most important risk factors for calcific aortic valve disease, significantly influencing both the severity and prevalence of valvular calcification, as well as atherosclerosis. The prevalence of both conditions increases with age. Even though the atherosclerotic process usually begins during childhood and eventually progresses to atheroma, the most significant increase in severity and prevalence of the disease has been observed after the age of 70, due to the accumulation of multiple pathological processes such as lipid deposition, progressive calcification, and chronic inflammation [1,3,4].

Another modifiable risk factor that also leads to valvular calcification and atherosclerosis is gender. The risk of atherosclerosis is higher in males compared to women, and several studies have shown that calcification is greater in men than women. The mechanism could be hormonal due to estrogen, which inhibits calcification in women, or sex-linked genetic factors [9].

A family history of aortic stenosis or atherosclerosis can increase the risk of developing the condition, especially in siblings. Although the genetic background of aortic valve calcification is still unknown, there could be an association between genetic predisposition to increased levels of lipoprotein(a) and plasma lipids. Nonetheless, genetic mutations like NOTCH1 mutations, lipoprotein metabolism genes, calcium signaling pathway genes, and runt-related transcription factor 2 (RUNX2) have been associated with valvular calcification [9].

The modifiable risk factors common to both aortic stenosis and atherosclerosis include high blood pressure, smoking, dyslipidemia, diabetes mellitus, kidney dysfunction, excess abdominal visceral fat, and a sedentary lifestyle [4].

There is a clear link between blood pressure and aortic valve stenosis, as well as blood pressure and atherosclerosis. The mechanical stress caused by elevated blood pressure on the aortic valve can speed up calcification at this site by damaging tissues and triggering inflammatory responses. It is also associated with worse outcomes in patients with preexisting valvular disease [10].

Cigarette smoking is one of the most common risk factors. Both active and passive smoking contribute to the process of aortic calcification and atherosclerosis. There is a dose–response relationship: long-term and high-dose smoking increases the risk of valvular disease. Calcification becomes significantly more advanced after the age of 45, and quitting smoking could lead to lower scores for age-related aortic valve calcification [11]. The mechanisms of aortic valve dysfunction in smokers may involve altered lipoprotein composition, increased oxidative stress, a proinflammatory state, and the release of free radicals [12].

Dyslipidemia, a condition highly prevalent in both aortic stenosis and atherosclerosis, is characterized by abnormal concentrations of lipids in the bloodstream. The accumulation of lipids as lipoprotein(a) and low-density lipoprotein (LDL) cholesterol contributes to valvular calcification and plaque buildup in arteries. Lipoprotein(a) is responsible for carrying oxidized phospholipids, which damage the valve tissue. Also, the contribution of LDL-cholesterol to the inflammatory process can lead to calcium deposition [6].

Type II diabetes mellitus is a significant metabolic risk factor for both valvular calcification and atherosclerosis, especially given its increasing global incidence. Elements as dysregulation of dipeptidyl peptidase-4 (DPP-4), insulin growth factor-1 (IGF-1), advanced glycation end products (AGEs), and insulin resistance increase the risk of developing severe aortic stenosis in individuals with diabetes mellitus [2]. The prevalence is higher in patients with diabetes mellitus, and the disease has a faster progression due to a proinflammatory state, oxidative stress, and altered cellular metabolism in valve tissues [2].

Advanced kidney dysfunction is significantly associated with both aortic calcification and atherosclerosis. As renal function declines, levels of minerals such as calcium and phosphorus increase, promoting calcification of blood vessels and heart valves, particularly the aortic valve. As the valve becomes more stenotic, blood flow to the kidneys is further reduced, thus worsening the renal dysfunction [13].

Similar to atherosclerosis, visceral adipose tissue is strongly associated with aortic stenosis, as it promotes the production of inflammatory cytokines and oxidative stress mediators. Additionally, adipose tissue accumulation can lead to metabolic disturbances such as hypertension, dyslipidemia, and features of atherosclerosis, all of which contribute to a proinflammatory state and promote calcium deposition in the aortic valve [14] or blood vessels.

Physical inactivity is a behavioral lifestyle factor that may lead to aortic valve calcification. Higher rates of calcium deposits in arteries have been found in individuals with low physical activity due to the inflammation and elevated blood pressure caused by low physical activity [1].

Atherosclerosis and valvular calcification are such complex processes determined by multiple interactions between many common risk factors, including age, male gender, genetic, metabolic, behavioral, or cardiovascular factors. Continuing to explore the mechanisms of the disease and the risk factors is mandatory for prevention and targeted early treatment.

## 4. Mechanisms

### 4.1. Aortic Stenosis and Atherosclerosis

#### 4.1.1. Shared Pathological Features

The association between calcific aortic valve disease and atherosclerosis has been recognized since 1946. Both conditions exhibit lipid accumulation, infiltration by inflammatory cells (macrophages, T lymphocytes), and calcific deposits. Early lesions in the aortic valve involve collagen disruption and lipid deposition, followed by progressive calcification. Osteopontin and tenascin—extracellular matrix proteins involved in mineralization and remodeling—are present in both atherosclerotic plaques and stenotic valves [15].

Inflammatory infiltrates in early calcific aortic valve disease (CAVD) include T cells and macrophages, which, together with elevated levels of apolipoprotein B and lipoprotein(a), as well as apolipoprotein E deficiency, contribute to lesion formation. These cells upregulate matrix metalloproteinases (MMPs) and their inhibitors, promoting extracellular matrix degradation and remodeling, a mechanism shared with atherogenesis. The expression of angiotensin-converting enzyme (ACE) and chymase in calcified valves is mediated by macrophages and mast cells and is linked to low-density lipoprotein (LDL) cholesterol, echoing similar pathways in coronary artery disease [6,8].

Aortic valve calcification correlates with calcifications of the coronary arteries, aorta, and mitral annulus. These findings are strongly associated with systemic atherosclerosis markers, such as carotid intima-media thickness (IMT). An IMT >1.2 mm in patients with aortic stenosis (AS) is predictive of concomitant coronary artery disease. Mitral annular calcification and valvular sclerosis are independent predictors of cardiovascular morbidity, mortality, and ischemic stroke [16].

#### 4.1.2. Differentiating Mechanisms from Atherosclerosis

While both diseases originate from an inflammatory stimulus, their pathophysiological evolution diverges. Atherosclerosis is driven by inflammation and predisposes individuals to acute events (e.g., plaque rupture, thrombosis), whereas CAVD progresses chronically due to calcification-induced leaflet stiffening and left ventricular outflow obstruction [17]. Imaging studies (e.g., Dweck et al.) revealed more intense calcification in stenotic valves compared to atheromatous aortic regions, but relatively less inflammation, indicating that calcification predominates in CAVD progression [8].

### 4.2. Aortic Stenosis: Pathogenesis

**Figure 1:**
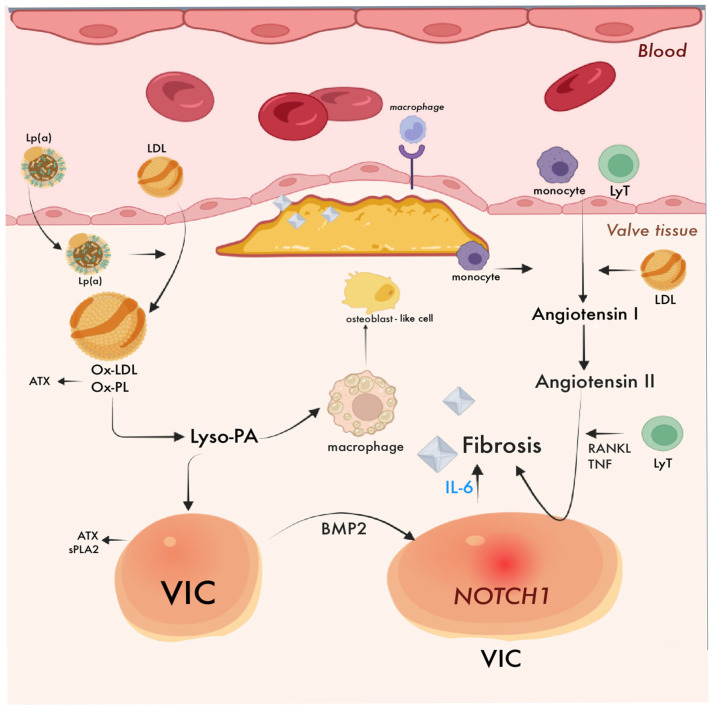
Pathophysiological pathways driving CAVS progression. TNF-α and signaling pathways such as NOTCH1 and BMP2, which drive pro-calcific gene expression. Additionally, angiotensin II enhances inflammation and may facilitate VIC activation via RANKL signaling. The net effect is a vicious cycle of inflammation, lipid oxidation, and osteogenic transformation, contributing to fibrotic and calcific remodeling of the valve [19].

The pathogenesis of calcific aortic valve stenosis (CAVS) progresses through two main phases: initiation and propagation. The initiation phase is primarily driven by inflammation and lipid accumulation, especially following endothelial injury [18]. This process promotes infiltration by inflammatory cells such as macrophages, T-lymphocytes, and mast cells, which in turn intensify oxidative stress, lipid oxidation, and early microcalcification. Microcalcifications further drive inflammation, apoptosis, and defective phagocytosis, contributing to lesion progression [5]. Figure 1 illustrates these mechanisms, summarizing the contribution of lipoprotein(a), oxidized lipids, inflammatory cells, and key signaling pathways to the progression of CAVS.

In contrast, the propagation phase relies less on inflammation and is characterized by fibrosis and accelerated calcification, leading to valvular stiffening, dysfunction, and altered hemodynamics. Key pro-fibrotic mechanisms include reduced nitric oxide bioavailability and activation of the renin–angiotensin system (RAS), particularly angiotensin II (AngII). Downregulation of the AngII type 2 receptor facilitates collagen deposition and promotes calcification [5,18].

Phenotypic switching of valvular interstitial cells (VICs) into osteoblast-like cells is a central event in disease progression. This osteogenic transformation is mediated by several signaling pathways, including NOTCH, RANK/RANKL/OPG (Receptor Activator of Nuclear Factor κB/RANK Ligand/osteoprotegerin), Wnt/β-catenin, and bone morphogenetic proteins (BMPs), particularly BMP-2, which induces osteogenic markers such as alkaline phosphatase and osteocalcin [20].

Matrix Gla-protein (MGP), a vitamin K-dependent inhibitor of calcification, regulates this process by both inhibiting BMP-2 signaling and binding directly to hydroxyapatite crystals, preventing their growth. MGP exists in two forms: active (carboxylated) and inactive (uncarboxylated). Activation requires vitamin K, and vitamin K antagonists (VKAs) such as warfarin disrupt this process, enhancing vascular and valvular calcification, as shown in animal and human studies [21].

As understanding of CAVS pathophysiology advances, new therapeutic targets emerge, including BMP pathways, the RANK/RANKL/OPG axis, and vitamin K-dependent mechanisms that may help slow or prevent disease progression [21].

As the understanding of CAVS pathophysiology deepens, several potential therapeutic targets emerge, including BMP signaling, the RANK/RANKL/OPG axis, and vitamin K-dependent pathways, which may help slow or prevent disease progression [22].

#### 4.2.1. Endothelial Dysfunction

The aortic valve consists of two types of cells: VICs (interstitial valve cells, such as fibroblasts, smooth muscle cells) on the inner layer and VECs (endothelial valve cells) forming the outer layer. When shear stress damages the outer layer (VECs), the inner cells (VICs) might secrete growth factors and molecules for osteogenic differentiation, leading to lipoprotein deposits. Moreover, VECs can also differentiate into mesenchymal cells (EndMT) that promote inflammation, proliferation, and extracellular matrix synthesis [21,23].

#### 4.2.2. Oxidative Stress and Lipid Deposits

One polymorphism in the Lp(a) encoding gene (LPA) is associated with AS. There is also evidence supporting the theory that PCSK9 (proprotein convertase subtilisin/kexin type 9) exacerbates valve calcification; loss of function of the gene encoding PCSK9 might reduce the risk of CAVS. PCSK9 inhibitors decrease the incidence of new or aggravated AS [17,24].

A total of 50% of patients with CAVD have concomitant coronary artery atherosclerotic disease [25]. High levels of circulating oxidized low-density lipoprotein (Ox-LDL) are associated with worse fibrocalcific remodeling of valvular tissue in calcific AS valves, with higher oxidized LDL content showing significantly increased density of inflammatory cells, expression of TNF-alpha, and tissue remodeling score [26]. High-density lipoprotein (HDL) mitigates atherogenesis by modulating oxidative and inflammatory responses in the arterial wall through delivering antioxidant enzymes, regulating lipid cargo, and activating the receptor-mediated signaling pathways, thereby opposing the pathogenic effects of apoB-containing lipoproteins and Lp(a). HDL lowers ROSs (reactive oxygen species) and lipid hydroperoxides, attenuates TNF-α–driven inflammatory signaling, and reprograms macrophages toward less inflammatory, non-foam cell phenotypes [27].

Recently, elevated lipoprotein(a) and OxPL-apoB (oxidized phospholipids associated with apolipoprotein B) levels have been associated with faster AS aggravation and the need for aortic valve replacement [28,29].

Patients with preexisting mild-to-moderate aortic stenosis (AS) who exhibit elevated levels of OxPL-apoB, OxPL-apo(a), and lipoprotein(a) experience a significantly accelerated progression of valvular disease and a higher incidence of requiring aortic valve replacement [30].

Lipoprotein(a) [Lp(a)] and oxidized phospholipids (OxPL) contribute to the development of CAVD by binding to exposed or damaged valve surfaces through strong lysine-binding sites on apolipoprotein(a) [apo(a)] [31]. In individuals with elevated levels of lipoprotein(a) enriched in OxPL, this lipoprotein can adhere firmly to the exposed valvular tissue. Subsequently, through its associated OxPL, it may promote chronic inflammation and calcification of valvular cells, thereby accelerating the progression of CAVD [32].

Oxidative stress, partly due to impaired antioxidant defenses and nitric oxide synthase dysfunction, promotes aortic valve calcification by inhibiting cGMP (cyclic guanosine monophosphate) signaling [33]. Ataciguat, a soluble guanylate cyclase activator that targets the enzyme’s oxidized form, has demonstrated in preclinical and early clinical studies the potential to reduce calcification and slow the progression of the disease [34].

#### 4.2.3. Inflammation and Fibrosis

Activated macrophages, monocytes, and proinflammatory mediators secrete TNF alpha, IL-1β (interleukin-1 beta), increasing also the extracellular matrix MMP1 and MMP2, resulting in damaged VECs [35].

Recent studies have shown that Piezo1 (a mechanoreceptor on the cell surface of the aortic valve) senses the stress and activates monocytes and inflammation, a process that does not occur after TAVI (Transcatheter Aortic Valve Implantation) [36].

Dipeptidyl peptidase 4 (DPP4) increases valvular calcification and progression of AS [37]. Inhibition of DPP-4 enzymatic activity by sitagliptin in a rabbit CAVD model resulted in significant improvements in aortic valve area, transaortic peak velocity, and maximal and mean pressure gradients after 12 weeks of treatment [38]. Thiazolidinediones (TZDs) also reduce the expression of advanced glycation end products (RAGE), promoting the potential anti-inflammatory effect [19]. Histological studies of stenotic aortic valves have linked the accumulation of AGEs to oxidative stress, inflammation, coagulation factor expression, and calcification [39]. In diabetic patients with severe AS, antidiabetic therapies may offer local valvular or myocardial benefits [40]. However, no current medical treatment has shown a survival advantage. Aortic valve replacement remains the only proven therapy, as diabetic patients face a higher postoperative risk than non-diabetics [41].

Evidence from small studies suggests that RAAS (renin-angiotensin-aldosterone system) inhibitors may have beneficial effects in aortic stenosis beyond blood pressure control [42]. These agents could interfere with fibrotic pathways in the valve and heart muscle, possibly delaying disease progression and improving structural cardiac adaptations [43].

#### 4.2.4. Differentiation and Osteogenic Calcification

VICs can turn into myofibroblasts and osteoblasts when activated by TGF1β [a process inhibited by FGF2 (fibroblast growth factor 2)], leading to the expression of cadherin 11 and inducing the production of myofibroblasts [44].

Aortic valvular calcification involves a complex, regulated process of biomineralization that mimics osteogenesis. When mineralization is induced via oxidative stress, DRP1 (dynamin-related protein 1) promotes human cardiovascular calcification by regulating osteogenic differentiation [45].

Studies have found an age-independent correlation between aortic calcification and osteoporosis in women, as there are common risk factors for both osteoporosis and CV disease, atherosclerotic calcification being positively associated with bone loss [46]. Inflammatory cytokines and lipid oxidation products promote CV calcification in parallel with atherosclerosis [47].

In the cardiovascular system, osteoclast-like cells can develop from both M1 and M2 macrophages; notably, high-density lipoprotein is recognized as a key modulator of macrophage polarization, influencing the balance between M1 and M2 states. Recent findings further indicate a wider spectrum of macrophage phenotypes beyond this classical dichotomy. Osteoclast-like cell function is modulated by vascular smooth muscle cells (VSMCs) and VICs, which are capable of acquiring an osteoblast-like phenotype [48]. VSMCs and VICs exhibit significant phenotypic plasticity, and under pathological stimuli such as inflammation, oxidative stress, or hyperphosphatemia, they can transdifferentiate into osteogenic-like cells, contributing to vascular and valvular calcification. These osteogenic cells produce RANKL and M-CSF, key factors that promote macrophage differentiation into osteoclast-like cells, a process also reproducible under in vitro conditions [49]. Therefore, Denosumab, a monoclonal antibody targeting RANKL, blocks its interaction with RANK, thereby mimicking osteoprotegerin (OPG) and potentially suppressing osteogenic differentiation within aortic valve tissue [50]. Denosumab and bisphosphonates are under investigation in the SALTIRE II trial for their potential to modulate calcification in AS [51,52].

Of note, diabetes may affect vascular calcification by promoting the release of osteoprogenitor cells from the bone marrow. Circulating progenitor cells from diabetic patients show a phenotypic drift toward a pro-calcific phenotype driven by inflammatory signals [53]. Prediabetic patients express the osteoblastic marker osteocalcin.

Supplementation with vitamin K represents a promising strategy to restore vascular vitamin K levels and enhance the inhibition of pathological calcification. In animal studies, particularly in rats, vitamin K supplementation has been shown to reverse warfarin-induced vascular calcification [54].

#### 4.2.5. Angiogenesis and Hemorrhage

The aortic valve is avascular, but in pathological cases such as rheumatic disease or calcific disease, neovascularization can occur by upregulating the VEGF and its receptors. The pathological accumulation of hemoglobin contributes to iron overload and oxidative stress, primarily via hemolytic processes and the extracellular release of free hemoglobin. Following erythrocyte lysis, hemoglobin is released into the extracellular space, where it undergoes autoxidation, resulting in the generation of ROSs, including superoxide anions and hydrogen peroxide. These ROSs promote further oxidative modifications of hemoglobin, yielding ferryl and oxyferryl intermediates that facilitate the liberation of free heme and labile iron. Non-transferrin-bound iron, in particular, produces hydroxyl radicals that exacerbate oxidative tissue damage [55].

#### 4.2.6. Shear Stress

Elevated blood pressure can intensify mechanical loading on the aortic valve leaflets during diastole by increasing the transvalvular pressure difference [56]. This heightened stress may contribute to microstructural damage, promote local inflammation, and impair endothelial integrity [57]. Over time, these changes may accelerate degenerative remodeling of the valve. Consequently, effective blood pressure control could offer protective effects beyond general cardiovascular risk reduction, potentially slowing the progression of aortic stenosis [58].

#### 4.2.7. Genetic Predisposition and Visceral Obesity

A predisposition determined by genetic factors that leads to elevated LDL cholesterol appears to be specifically associated with the initiation of calcific and atherosclerotic changes in the aortic valve, rather than variations in HDL cholesterol or triglyceride levels [59]. Experimental studies on animal models have indicated that interventions aiming to reduce plasma cholesterol may slow down the advancement of aortic valve disease [56].

In cases of visceral obesity, patients commonly exhibit a lipid profile characterized by reduced HDL and increased concentrations of LDL, especially the small, dense LDL particles [6]. These are more prone to penetrate the aortic valve tissue and become oxidized, a process facilitated by the decreased anti-inflammatory protection offered by low HDL levels [60]. This promotes an inflammatory response, attracting immune cells to the valve. Macrophages exposed to oxidized LDL begin secreting inflammatory mediators like tumor necrosis factor-alpha (TNF-α), which can trigger the transformation of valve myofibroblasts into cells with osteoblastic features [61]. Such processes of osteogenic differentiation in the aortic valve—whether in standard CAVD or forms associated with bicuspid aortic valves—are also influenced by the Lrp5 (Low-Density Lipoprotein Receptor-Related Protein 5)/Wnt3 signaling cascade [62].

### 4.3. The Role of Plasma Biomarkers in Moderate-to-Severe Aortic Stenosis

In patients with moderate-to-severe AS, several circulating biomarkers offer insight into disease severity and progression. N-terminal pro-B-type natriuretic peptide (NT-proBNP), a marker of myocardial wall stress, tends to rise significantly even in asymptomatic individuals, reflecting early hemodynamic burden despite preserved ventricular function [63,64]. Higher NT-proBNP concentrations are associated with impaired coronary flow reserve and worse clinical outcomes, including hospitalizations, surgical interventions, and mortality [19].

Beyond NT-proBNP, other biomarkers highlight the multifactorial nature of AS. Elevated levels of tissue factor (TF) have been found in heavily calcified valve tissue, suggesting a role in promoting inflammation, thrombosis, and angiogenesis. Inflammatory mediators such as lipoprotein-associated phospholipase A_2_ (Lp-PLA_2_) are also elevated and appear to correlate with disease severity [65]. Adipocytokines, particularly low levels of adiponectin and leptin, may contribute to valvular remodeling and are more commonly observed in advanced AS [66]. Furthermore, abnormalities in coagulation, such as acquired von Willebrand syndrome and shear stress-induced thrombosis, emphasize the complex interactions between mechanical forces, vascular biology, and systemic inflammation in the pathogenesis of AS [65].

## 5. Pharmaceutical Therapies Targeting Both Atherosclerosis and Aortic Stenosis

Atherosclerosis and aortic valve stenosis are progressive pathologies marked by shared mechanistic underpinnings, including lipid infiltration, chronic inflammation, oxidative stress, and active osteogenic calcification [6]. These conditions, though anatomically distinct, exhibit parallel disease pathways at the molecular and cellular levels, particularly in the activation of pro-calcific signaling in response to endothelial dysfunction, lipid oxidation, and inflammatory cell infiltration [21].

This chapter reviews and expands upon the current pharmaceutical strategies, specifically PCSK9 (proprotein convertase subtilisin/kexin type 9) inhibitors and lipoprotein(a)-lowering RNA-based agents, with mechanistic insight drawn from recent advances in our understanding of calcific aortic valve stenosis progression—Table 1.

### 5.1. PCSK9 Inhibitors (Evolocumab, Alirocumab)

PCSK9 inhibitors are monoclonal antibodies that block the PCSK9-mediated breakdown of low-density lipoprotein receptors, leading to enhanced clearance of low-density lipoprotein cholesterol (LDL). Clinical studies have shown they can lower LDL levels by 50–60% and lipoprotein(a) (Lp(a)) levels by 20–30%. The FOURIER trial, involving 27,564 patients with atherosclerotic cardiovascular disease, indicated that evolocumab significantly reduced major adverse cardiovascular events (MACEs) by 15% over an average follow-up of 2.2 years [67]. Similarly, the ODYSSEY OUTCOMES trial found that alirocumab reduced the risk of recurrent ischemic events in patients with recent acute coronary syndrome [68].

In addition to lowering lipid levels, PCSK9 inhibitors may provide direct vascular and valvular advantages. A post hoc analysis of the FOURIER trial revealed that patients receiving evolocumab had a significantly reduced risk of developing aortic stenosis during the follow-up period [68]. PCSK9 is found in calcified aortic valves and is linked to the activation of valvular interstitial cells and the formation of calcific nodules. Research in experimental models has shown that PCSK9 enhances osteogenic differentiation by upregulating BMP-2, runt-related transcription factor 2 (RUNX2), and alkaline phosphatase—key mediators in the calcification process [65,69]. Inhibition of PCSK9 in murine models has demonstrated a decrease in vascular calcification, oxidative stress, and apoptosis in vascular smooth muscle cells [69]. These results imply that PCSK9 inhibitors may play a role in slowing the progression of calcific aortic valve stenosis (CAVS), though additional validation in clinical trials is needed.

### 5.2. Lp(a)-Lowering RNA-Based Therapies

Elevated lipoprotein(a) is a well-established risk factor for both atherosclerosis and CAVS. Lp(a) is a unique lipoprotein particle consisting of a low-density lipoprotein (LDL)-like core containing apolipoprotein B-100 (apoB), to which apolipoprotein(a) (apo(a)) is covalently attached via a disulfide bond. Apo(a) is characterized by a distinctive structure containing multiple kringle IV repeats, which confer its unique biochemical properties. Lp(a) particles are highly enriched in oxidized phospholipids (OxPL), which promote endothelial dysfunction, inflammation, and calcification in vascular and valvular tissues [70]. The covalent linkage of apo(a) to apoB stabilizes Lp(a) and influences its atherogenic and pro-calcific effects. Genetic studies have confirmed a causal relationship between elevated Lp(a) levels and both coronary artery disease and aortic valve stenosis [71].

Therapeutic agents such as pelacarsen (an antisense oligonucleotide), olpasiran, and lepodisiran (small interfering RNAs) specifically target the hepatic synthesis of apo(a), leading to profound reductions in circulating Lp(a) levels, up to 98% [72,73,74]. The ongoing Lp(a) HORIZON trial is evaluating whether pelacarsen can reduce cardiovascular events in high-risk individuals with elevated Lp(a) [9]. Olpasiran and lepodisiran have demonstrated durable reductions in Lp(a) for up to 48 weeks following a single injection [73,74].

In patients with aortic stenosis (AS), elevated Lp(a) levels are associated with faster disease progression and an increased need for valve replacement [75]. Histological studies have shown colocalization of Lp(a), OxPL, and oxidized LDL within stenotic aortic valve tissue [75]. Additionally, lipoprotein-associated phospholipase A_2_ (Lp-PLA_2_), an enzyme carried by Lp(a), has been implicated in amplifying inflammatory responses in valve interstitial cells, thereby enhancing osteogenic signaling and calcification [71,76]. These data support the hypothesis that potent Lp(a) lowering could attenuate both atherosclerotic and valvular calcification processes.

PCSK9 inhibitors and Lp(a)-lowering RNA-based therapies represent two promising pharmacological strategies with therapeutic potential in both atherosclerosis and calcific aortic valve stenosis. While PCSK9 inhibitors have already demonstrated cardiovascular event reduction and possibly reduce the incidence of aortic stenosis, RNA-based therapies offer a novel approach targeting the causal role of Lp(a) in these diseases. Ongoing clinical trials will clarify whether these agents can modify disease progression and reduce the need for invasive valve interventions [59].

### 5.3. Renin–Angiotensin–Aldosterone System (RAAS) Inhibitors

In addition to PCSK9 inhibitors and Lp(a)-targeted RNA-based agents, several other pharmacologic strategies, both established and emerging, are being explored for their ability to modulate shared pathogenic pathways in atherosclerosis and CAVS. These include therapies targeting inflammation, oxidative stress, fibrotic remodeling, and calcification, hallmarks common to both vascular and valvular disease [77].

Among these, inhibition of the renin–angiotensin–aldosterone system (RAAS) has garnered particular interest due to its central role in cardiovascular remodeling. Angiotensin II (AngII), the principal effector of RAAS, is a key mediator of oxidative stress, endothelial dysfunction, and fibroblast activation. In atherosclerosis, AngII promotes vascular inflammation, smooth muscle cell migration, and destabilization of atherosclerotic plaques [78]. Pharmacologic RAAS blockade with angiotensin-converting enzyme (ACE) inhibitors or angiotensin receptor blockers (ARBs) is widely used in clinical cardiology to treat hypertension and heart failure. Observational data suggest that these agents may provide benefit in CAVS beyond blood pressure control [79,80]. A population-based study involving over 2000 patients from Scotland with AS found that RAAS inhibitor use was associated with reduced rates of all-cause mortality and cardiovascular events over an average follow-up of 4.2 years [81].

Although randomized controlled trials are still needed to definitively establish the role of RAAS blockade as a disease-modifying treatment in CAVS, current mechanistic evidence and clinical observations suggest that these agents may beneficially influence both atherosclerotic and valvular pathways, particularly in patients with concomitant hypertension or left ventricular hypertrophy [77].

### 5.4. DPP-4 Inhibitors

Dipeptidyl peptidase-4 (DPP-4) is a serine protease with multiple biological roles, including degradation of incretins, regulation of immune cell activity, and modulation of inflammation and fibrosis. DPP-4 inhibitors are a class of orally administered, small-molecule drugs designed to selectively block the enzymatic activity of DPP-4. By preventing the cleavage of glucagon-like peptide-1 (GLP-1) and glucose-dependent insulinotropic polypeptide (GIP), these agents prolong incretin activity, enhancing glucose-dependent insulin secretion and reducing glucagon release. The most widely used DPP-4 inhibitors include sitagliptin, linagliptin, saxagliptin, alogliptin, and evogliptin, which share similar pharmacological targets but differ in their pharmacokinetic properties and tissue distribution.

Beyond their glucose-lowering effects, DPP-4 inhibitors have been implicated in processes relevant to both atherosclerosis and CAVS, particularly in individuals with type 2 diabetes mellitus, a population prone to overlapping vascular and valvular pathologies [82]. Experimental evidence has shown that increased DPP-4 activity contributes to the progression of CAVS by promoting valvular inflammation, fibrosis, and osteogenic differentiation of valvular interstitial cells. In a preclinical model, sitagliptin significantly reduced valvular calcification and fibrosis, improving hemodynamic parameters and slowing the progression of AS. These effects were attributed to reduced inflammatory cell infiltration and decreased expression of osteogenic markers in the aortic valve tissue [37]. Furthermore, treatment with evogliptin, a DPP-4 inhibitor with high cardiac tissue bioavailability, was associated with inhibition of fibrotic remodeling and calcific nodule formation in valvular tissue, indicating a direct impact on the pathophysiological mechanisms driving CAVS [22].

The anti-atherosclerotic properties of DPP-4 inhibitors are also supported by multiple molecular and in vivo studies. Linagliptin was shown to attenuate the formation of macrophage-derived foam cells and to suppress oxidized LDL-induced inflammation by downregulating the nuclear factor kappa-light-chain-enhancer of activated B cells (NF-κB) pathway and inhibiting activation of the NOD- (nucleotide-binding oligomerization domain), LRR- (leucine-rich repeat), and pyrin domain-containing protein 3 (NLRP3) inflammasome in THP-1 macrophages. These effects translated into reduced secretion of pro-inflammatory cytokines such as interleukin-1 beta (IL-1β) and tumor necrosis factor alpha (TNF-α), both implicated in plaque progression and instability [82]. Complementary findings from a comprehensive review of preclinical models demonstrated that various DPP-4 inhibitors reduce vascular oxidative stress, monocyte recruitment, and vascular smooth muscle cell proliferation—mechanisms pivotal to early and late atherosclerotic lesion development [83]. In vivo imaging studies have confirmed these findings. In a murine model of atherosclerosis and type 2 diabetes, DPP-4 inhibition led to a significant reduction in arterial inflammation as assessed by 18F-FDG uptake. This anti-inflammatory response was accompanied by downregulation of macrophage activity in the arterial wall and decreased systemic inflammatory markers, providing functional evidence for vascular protection [84]. Similarly, sitagliptin was shown to reduce apoptosis of vascular smooth muscle cells (VSMCs) in diabetic ApoE-deficient mice, a mechanism linked to plaque vulnerability. Histological analyses confirmed smaller lesion areas and reduced necrotic cores, with preserved fibrous cap integrity in treated animals, suggesting enhanced plaque stability [85].

Altogether, these findings highlight the pleiotropic benefits of DPP-4 inhibitors beyond glycemic control. Their anti-inflammatory, anti-fibrotic, and anti-calcific effects offer a potential disease-modifying strategy for patients with coexisting atherosclerosis and AS, especially in the diabetic population. However, further clinical trials are needed to validate these results in broader patient cohorts and to assess their impact on hard cardiovascular endpoints [83].

### 5.5. Vitamin K Supplementation

Vitamin K is essential for the gamma-carboxylation and subsequent activation of matrix Gla-protein (MGP), a potent endogenous inhibitor of vascular and valvular calcification. In its inactive, uncarboxylated form, MGP loses the ability to bind and inhibit bone morphogenetic proteins such as bone morphogenetic protein-2 (BMP-2), which promotes osteogenic differentiation and hydroxyapatite deposition within vascular and valvular structures. This mechanism underlies the progressive mineralization seen in CAVS and atherosclerosis, particularly in patients with chronic kidney disease or diabetes mellitus [54].

Pharmacologic inhibition of vitamin K recycling through agents like warfarin has been shown to accelerate vascular and valvular calcification. This effect is largely due to the accumulation of inactive, uncarboxylated MGP, which fails to regulate mineralization pathways. Conversely, vitamin K supplementation—particularly with menaquinones (vitamin K2)—has demonstrated the capacity to restore MGP activity, suppress BMP signaling, and reduce mineral deposition in preclinical models. Recent clinical investigations have sought to evaluate the translational potential of vitamin K supplementation in individuals at risk of cardiovascular calcification [46]. A randomized double-blind placebo-controlled trial investigating combined supplementation with vitamin K2 and D3 in patients with aortic valve calcification failed to show a significant reduction in calcification progression over two years, although subgroup analysis suggested possible benefit in individuals with lower baseline vitamin K status [86]. Another study conducted in individuals with type 2 diabetes mellitus found that six months of vitamin K supplementation increased serum calcification resistance, as measured by T50 time, suggesting a more favorable milieu for inhibiting calcium-phosphate precipitation in plasma [87]. Additionally, a systematic review of controlled trials examining vitamin K supplementation for cardiovascular disease prevention has highlighted the potential of vitamin K2 to slow subclinical progression of arterial stiffness and coronary calcification, though results remain heterogeneous and inconclusive regarding hard outcomes [88]. These findings suggest a biologically plausible and potentially therapeutic role for vitamin K in reducing both vascular and valvular calcification. However, current evidence from interventional trials remains insufficient to support routine supplementation. Further research is needed to determine the optimal dose, formulation, and patient populations most likely to benefit.

### 5.6. Denosumab (Anti-RANKL Monoclonal Antibody)

The receptor activator of nuclear factor kB/receptor activator of nuclear factor kB ligand/osteoprotegerin (RANK/RANKL/OPG) signaling axis plays a central role in bone metabolism and is increasingly recognized as a key contributor to pathological calcification in cardiovascular tissues. In CAVS, valvular interstitial cells (VICs) exposed to pro-inflammatory and osteogenic stimuli express RANKL, which promotes their transdifferentiation into osteoblast-like cells capable of depositing calcium within the valvular extracellular matrix. Simultaneously, infiltrating macrophages and T lymphocytes contribute to this process by secreting RANKL, thus amplifying osteogenic signaling in the valve microenvironment [50].

Denosumab is a fully human monoclonal antibody that binds to RANKL and prevents its interaction with its receptor, RANK, mimicking the natural decoy receptor OPG. Originally developed and approved for the treatment of osteoporosis, denosumab has attracted interest in cardiovascular research due to its potential to inhibit ectopic calcification. The SALTIRE II trial investigated the effect of denosumab on aortic valve calcification and demonstrated that, although the primary endpoint of halting disease progression was not conclusively met, favorable trends suggested slowed calcific progression in selected subgroups, particularly in comparison with alendronate [89,90]. A secondary analysis of the same trial also explored its effects on vascular calcification, showing modest attenuation of progression in the aortic root and ascending aorta [89].

Beyond its anti-calcific potential, emerging data suggest that denosumab may exert favorable effects on lipid metabolism, inflammation, and the osteogenic signaling cascades involved in both valvular and vascular pathology. A recent study highlighted denosumab’s potential in modulating lipid profiles and reducing inflammation-driven metabolic disturbances, suggesting broader cardiometabolic implications of RANKL inhibition [91]. Given the involvement of RANKL signaling in both vascular atherosclerosis and valvular stenosis, denosumab emerges as a promising candidate for dual-action therapy targeting shared mechanisms of pathological calcification in these interconnected diseases.

### 5.7. Statins—A Cautionary Note

Statins are widely prescribed for the management of atherosclerotic cardiovascular disease due to their proven efficacy in reducing LDL cholesterol, improving endothelial function, and exerting anti-inflammatory effects that contribute to plaque stabilization. However, their benefit does not appear to extend to the progression of calcific aortic valve stenosis. Several large randomized trials, including SALTIRE I and ASTRONOMER, failed to demonstrate any significant slowing of valvular calcification or improvement in valve hemodynamics with statin therapy in patients with mild-to-moderate aortic stenosis, despite effective lipid lowering [7,77].

One potential explanation lies in the differing pathophysiology between atherosclerosis and CAVS. While lipid accumulation and inflammation are early contributors to both diseases, the progression of CAVS is increasingly dominated by osteogenic and fibrotic processes that are less dependent on cholesterol levels. Furthermore, statins may paradoxically increase plasma concentrations of Lp(a), a pro-calcific and pro-inflammatory lipoprotein strongly associated with CAVS progression. This unintended effect may negate or even counteract any potential benefits from LDL-C reduction in the valvular context [7].

Although statins remain indispensable in patients with coexisting coronary artery disease or elevated LDL-C, current guidelines do not recommend their use solely to halt AS progression in patients without dyslipidemia. Moreover, evidence suggests that their role should be considered in the broader therapeutic context, possibly in combination with agents that more directly target fibrotic and osteogenic signaling cascades [77].

### 5.8. NOX2 Inhibition—Celastrol

Oxidative stress plays a pivotal role in the pathogenesis of both atherosclerosis and CAVS, promoting lipid peroxidation, endothelial dysfunction, and osteogenic differentiation of VSMCs and valvular interstitial cells (VICs). Among the major enzymatic sources of reactive oxygen species (ROSs) in cardiovascular tissues is NADPH oxidase 2 (NOX2), which is upregulated in both atherosclerotic lesions and stenotic valves [92].

Celastrol, a bioactive triterpenoid derived from *Tripterygium wilfordii*, acts as a selective NOX2 inhibitor with potent anti-inflammatory and antioxidative properties. Preclinical studies demonstrate that celastrol attenuates the expression of pro-inflammatory cytokines (TNF-α, IL-1β), suppresses ROS production, and downregulates osteogenic markers such as BMP-2 and RUNX2 in VICs and VSMCs [93,94]. These actions contribute to the inhibition of early osteogenic reprogramming and valvular calcification. In atherosclerosis models, celastrol has shown efficacy in reducing plaque size, foam cell formation, and macrophage infiltration [95,96,97]. Furthermore, innovative delivery systems, such as celastrol-loaded recombinant HDL nanoparticles, have enhanced its therapeutic potential while reducing systemic toxicity [94]. Specifically regarding aortic stenosis, celastrol has been shown to suppress NOX2-mediated ROS generation and calcification in cultured human VICs and animal models [92]. This mechanistic insight positions celastrol as a candidate for disease-modifying therapy in CAVS, though human studies are still lacking.

While celastrol remains in the experimental phase, its dual vascular and valvular protective effects, via NOX2 inhibition and downstream suppression of osteogenic and inflammatory signaling, mark it as a promising therapeutic lead for CAVS and atherosclerosis [98].

### 5.9. Soluble Guanylate Cyclase (sGC) Activators—Ataciguat

The nitric oxide (NO)–soluble guanylate cyclase (sGC)–cyclic GMP (cGMP) pathway plays a critical role in preserving endothelial integrity, regulating vascular tone, and suppressing fibrotic and osteogenic processes. In both atherosclerosis and calcific aortic valve stenosis, elevated oxidative stress depletes NO bioavailability and oxidizes the heme moiety of sGC, rendering it inactive. This interruption in cGMP signaling facilitates inflammation, calcification, and vascular remodeling [34].

Ataciguat is an oral activator of the heme-free form of sGC, capable of restoring cGMP synthesis even in the absence of NO signaling. By bypassing NO dependency, ataciguat reactivates downstream vasoprotective pathways, leading to vasodilation, inhibition of vascular and valvular smooth muscle cell proliferation, and repression of osteogenic differentiation in VICs and VSMCs [34]. These mechanisms offer a dual advantage in slowing both vascular and valvular degeneration. In a recent randomized controlled trial, ataciguat demonstrated a favorable safety profile and potential efficacy in reducing the progression of aortic valve calcification in patients with mild-to-moderate CAVS, as evidenced by decreased ^18F-NaF PET uptake and stabilized echocardiographic indices [34]. In parallel, sGC activation has been shown to counteract atherosclerotic plaque development by modulating platelet function, reducing vascular inflammation, and improving arterial compliance [99].

Taken together, these findings suggest that ataciguat represents a promising therapeutic strategy that addresses both CAVS and atherosclerosis through the restoration of redox-impaired sGC signaling. Ongoing clinical studies are expected to elucidate its long-term impact on cardiovascular outcomes.

### 5.10. Cadherin-11 Blockade—SYN0012

Cadherin-11 (CDH11) is a mesenchymal cell-specific adhesion molecule involved in intercellular mechanical coupling and profibrotic signaling. In calcific aortic valve disease, upregulation of cadherin-11 in VICs is a hallmark of pathological activation, promoting transformation into myofibroblast- and osteoblast-like phenotypes. This molecular reprogramming facilitates extracellular matrix remodeling, increased tissue stiffness, and calcific nodule formation [100,101,102].

SYN0012, a monoclonal antibody targeting cadherin-11, has shown efficacy in preclinical models by preventing valvular fibrosis and mineralization. Mechanistically, it disrupts cadherin-11-mediated mechanotransduction and adhesive interactions critical for VIC activation and calcification [103,104]. Cadherin-11 overexpression also plays a pathogenic role in vascular remodeling within atherosclerotic plaques, promoting fibrotic thickening and potentially destabilizing advanced lesions [105]. Notably, cadherin-11 blockade has been shown to reduce post-infarction myocardial fibrosis, indicating broader antifibrotic properties across cardiovascular tissues [106]. Given its role at the intersection of inflammation, fibrosis, and calcification, cadherin-11 inhibition with SYN0012 represents a promising therapeutic approach with potential applicability in both CAVS and atherosclerotic vascular disease.

### 5.11. Notch1 Stabilizers—XCT790

The Notch1 signaling pathway is a key regulator of cellular differentiation and homeostasis in cardiovascular tissues. In valvular interstitial cells, Notch1 suppresses osteogenic transformation by inhibiting the expression of key calcification mediators such as RUNX2 and osteocalcin. Genetic loss-of-function mutations in Notch1 are strongly associated with early-onset calcific aortic valve disease, underscoring its protective role in maintaining valve integrity [107,108,109].

XCT790, a pharmacologic modulator initially developed as an ERRα inverse agonist, has been shown to stabilize Notch1 protein levels and enhance its signaling activity in preclinical settings. By preventing proteasomal degradation of Notch1, XCT790 attenuates the osteogenic switch in VICs, reducing calcific nodule formation. Furthermore, Notch1 is also involved in maintaining vascular smooth muscle cell plasticity and repressing vascular calcification, suggesting dual protective effects in both valvular and arterial pathologies [110].

Although currently limited to exploratory and animal studies, pharmacologic activation or stabilization of Notch1 presents a highly specific and genetically informed strategy to counteract pathologic calcification, particularly in individuals with Notch1 insufficiency or mutations.

### 5.12. P2Y2 Receptor Agonists

Purinergic signaling, particularly through P2Y2 receptors, has emerged as a significant modulator of inflammation, fibrosis, and calcification in cardiovascular tissues. P2Y2 is a G protein-coupled receptor activated by extracellular adenosine triphosphate (ATP) and uridine triphosphate (UTP), and is expressed in endothelial cells, VSMCs and VICs [111].

Agonism of P2Y2 receptors exerts anti-inflammatory and anti-calcific effects by inhibiting TNF-α signaling pathways, reducing matrix metalloproteinase (MMP) activity, and downregulating osteogenic transcription factors such as RUNX2 and osteocalcin [112,113]. In VICs, this signaling prevents myofibroblastic activation and suppresses calcific nodule formation, especially in the early inflammatory stages of aortic stenosis [114]. Moreover, in atherosclerosis models, P2Y2 activation can reduce foam cell formation and VSMCs proliferation, thereby stabilizing plaques and limiting vascular remodeling [115].

Despite limited clinical data, preclinical studies support P2Y2 receptor agonists as a promising therapeutic strategy for mitigating both valvular and vascular degeneration. Their pleiotropic effects across endothelial and mesenchymal cell types highlight the translational potential of targeting purinergic signaling in calcific cardiovascular disease [112].

### 5.13. Conclusions and Translational Perspectives

The growing recognition of shared molecular pathways underlying atherosclerosis and calcific aortic valve stenosis has catalyzed the development of integrated therapeutic strategies targeting lipid metabolism, inflammation, oxidative stress, and osteogenic signaling (Table 1). Traditional agents such as PCSK9 inhibitors and Lp(a)-lowering RNA-based therapies have already demonstrated robust lipid-lowering effects and promising reductions in cardiovascular events. Their potential impact on valvular calcification, particularly through modulation of oxidized phospholipids and osteogenic markers, places them at the forefront of translational interest.

Beyond lipid regulation, a wave of emerging therapies offers targeted modulation of calcific and fibrotic pathways relevant to both vascular and valvular compartments. Agents such as NOX2 inhibitors, sGC activators, and Notch1 stabilizers aim to intercept redox imbalance and maladaptive differentiation of smooth muscle and interstitial cells—hallmarks of both diseases. In parallel, therapies repurposed from other indications, including DPP-4 inhibitors, RAAS blockers, and vitamin K supplementation, may offer incremental structural benefits through anti-inflammatory, anti-fibrotic, or anti-calcific effects. A unifying feature among these strategies is their mechanistic focus on early pathobiologic events—endothelial dysfunction, immune cell recruitment, and matrix remodeling—providing a rationale for initiating pharmacologic intervention before irreversible structural damage occurs. As demonstrated in ongoing and completed trials (e.g., SALTIRE II, HORIZON-Lp(a), and phase II studies with ataciguat), pharmacologic modulation of disease trajectory may eventually complement or delay invasive interventions such as valve replacement.

The road to clinical translation, however, demands well-designed randomized trials that consider the temporal overlap, phenotypic diversity, and comorbid burden of patients with atherosclerosis and CAVS. Future therapeutic frameworks may need to integrate biomarker-guided stratification, imaging-based monitoring of disease progression, and combined endpoints reflecting both vascular and valvular health. Ultimately, a tailored, mechanism-driven pharmacologic paradigm may reshape the therapeutic landscape of calcific cardiovascular disease, extending benefit across anatomical boundaries and disease stages.

## Figures and Tables

**Table 1:** Pharmaceutical therapies targeting both atherosclerosis and aortic stenosis.

Therapeutic Class/Agent	Mechanism of Action	Targets	Translational Notes
PCSK9 inhibitors/Lp(a)-targeted RNA therapies	Lower LDL-C and Lp(a) Reduce oxidized lipid-driven inflammation	Lipid metabolism, oxidized phospholipids	Effective in atherosclerosis Lp(a) lowering may delay CAVS progression
DPP-4 inhibitors (e.g., sitagliptin, evogliptin)	Anti-inflammatory, anti-oxidative Suppress valvular calcification via IGF-1/BMP-2 modulation	VICs, macrophages, and endothelium	Clinical data suggest slower AS progression in diabetics
Vitamin K (K1, K2)	Activates matrix Gla protein (MGP) Inhibits calcification	VICs, vascular smooth muscle cells	Safe, widely available Ongoing trials in CKD and elderly populations
Denosumab (anti-RANKL mAb)	Blocks RANKL–RANK interaction Inhibits osteogenic signaling and calcification	Osteoclast-like VICs, macrophages	Evaluated in SALTIRE II trial-modest reduction in valvular and vascular calcification
NOX2 inhibitors (e.g., celastrol)	Reduces oxidative stress and inflammatory cytokines Inhibits osteogenic markers (BMP-2, RUNX2)	VICs, VSMCs, macrophages	Dual benefit in atherosclerosis and CAVS in preclinical studies
sGC activators (e.g., ataciguat)	Restore cGMP signaling independent of NO Reduce fibrosis, calcification, and endothelial dysfunction	Endothelium, VICs, VSMCs	Phase II trial shows slowed AS calcification Benefits are also in arterial stiffness
Cadherin-11 inhibitors (e.g., SYN0012)	Blocks cell–cell adhesion and mechanotransduction Reduces VIC activation and fibrosis	Myofibroblasts, activated VICs	Experimental Prevents valvular and vascular fibrosis in preclinical models
Notch1 stabilizers (e.g., XCT790)	Maintains Notch1 signaling Inhibits the osteogenic transformation of VICs and VSMCs	VICs, VSMCs, developmental pathways	Promising in genetically predisposed models of early-onset CAVS
P2Y2 receptor agonists	Anti-inflammatory and anti-calcific Suppresses TNF-α, MMPs, and osteogenic transcription factors	VICs, VSMCs, endothelium	Emerging strategy Stabilizes plaques and reduces VIC calcification
RAAS inhibitors	Reduce fibrosis and inflammation Improve endothelial function	VSMCs, endothelium	Widely used May provide structural benefit beyond BP control
Statins (context-dependent)	Lower LDL-C, anti-inflammatory in atherosclerosis May increase Lp(a) and not benefit CAVS	Lipid metabolism, systemic inflammation	Strong role in CAD Not recommended for isolated AS without hyperlipidemia

## Data Availability

The raw data supporting the conclusions of this article will be made available by the authors on request.

## Abbreviations

ACE	angiotensin-converting enzyme
AGEs	advanced glycation end products
ANGII	angiotensin II
ARBs	angiotensin receptor blockers
Apo(a)	apolipoprotein(a)
ApoB	apolipoprotein B-100
AS	aortic stenosis
ATP	adenosine triphosphate
BMP-2	bone morphogenetic protein-2
BP	blood pressure
CAVD	calcific aortic valve disease
CAVS	calcific aortic valve stenosis
CDH11	cadherin-11
cGMP	cyclic guanosine monophosphate
DPP-4	dipeptidyl peptidase-4
DRP1	dynamin-related protein 1
EndMT	mesenchymal cells
FGF2	Fibroblast Growth Factor 2
GIP	glucose-dependent insulinotropic polypeptide
GLP-1	glucagon-like peptide-1
HDL	high-density lipoprotein
IGF-1	insulin growth factor-1
IL-1β	interleukin-1 beta
IMT	intima-media thickness
LDL	low-density lipoprotein
Lp(a)	lipoprotein(a)
Lp-PLA_2_	lipoprotein-associated phospholipase A_2_
Lrp5	Low-Density Lipoprotein Receptor-Related Protein 5
LRR	leucine-rich repeat
MACE	major adverse cardiovascular events
MGP	matrix Gla-protein
MMPs	matrix metalloproteinases
NF-κB	nuclear factor kappa-light-chain-enhancer of activated B cells
NLRP3	NOD-, LRR-, and pyrin domain-containing protein 3
NADPH	nicotinamide adenine dinucleotide phosphate
NO	nitric oxide
NOD	nucleotide-binding oligomerization domain
NOX2	NADPH oxidase 2
OPG	osteoprotegerin
Ox-LDL	oxidized low-density lipoprotein
OxPL-apoB	oxidized phospholipids associated with apolipoprotein B
OxPL	oxidized phospholipids
PCSK9	proprotein convertase subtilisin/kexin type 9
RAAS	renin-angiotensin-aldosterone system
RANK	receptor activator of nuclear factor kB
RANKL	receptor activator of nuclear factor kB ligand
ROS	reactive oxygen species
RUNX2	runt-related transcription factor 2
sGC	soluble guanylate cyclase
TAVI	transcatheter aortic valve implantation
TNF-α	tumor necrosis factor alpha
TZDs	thiazolidinediones
UTP	uridine triphosphate
VECs	endothelial valve cells
VICs	valvular interstitial cells
VKAs	vitamin K antagonists
VSMCs	vascular smooth muscle cells
